# Validation and Factor Analysis of the Japanese Version of the Highs Scale in Perinatal Women

**DOI:** 10.3389/fpsyt.2018.00269

**Published:** 2018-06-28

**Authors:** Aya Yamauchi, Takashi Okada, Masahiko Ando, Mako Morikawa, Yukako Nakamura, Chika Kubota, Masako Ohara, Satomi Murase, Setsuko Goto, Atsuko Kanai, Norio Ozaki

**Affiliations:** ^1^Department of Psychiatry, Nagoya University Graduate School of Medicine, Nagoya, Japan; ^2^Center for Advanced Medicine and Clinical Research, Nagoya University Hospital, Nagoya, Japan; ^3^Liaison Medical Marunouchi, Nagoya, Japan; ^4^Goto Setsuko Ladies Clinic, Nagoya, Japan; ^5^Graduate School of Education and Human Development, Nagoya University, Nagoya, Japan

**Keywords:** mood symptoms, pregnancy, postpartum, hypomania, depression

## Abstract

**Background:** The Highs scale has been developed to evaluate hypomanic symptoms in the first postpartum week. However, it has not been elucidated whether this scale is also applicable to pregnant women. To address this issue, we confirmed the factor structure, reliability, and validity of the Japanese version of the Highs scale for pregnant and postpartum women.

**Methods:** 418 women provided effective responses to both the Highs scale and the Edinburgh Postnatal Depression Scale (EPDS) during early pregnancy (before week 25), late pregnancy (around week 36), at 5 days and at 1 month after delivery. Subjects were randomly divided into two groups, and exploratory and confirmatory factor analyses were performed for each group. Cronbach's alpha was calculated and the correlation of the Highs scale with EPDS was analyzed. The correlation between the subscales was analyzed at four time points, and the correlation of subscales between the four time points was confirmed.

**Results:** This scale was found to have the two-factor structure with elation and agitation subscales. The two subscales had reasonable internal consistency at all time points (Cronbach's alpha range: Factor 1, 0.696–0.758; Factor 2, 0.553–0.694). The overall scale had reasonable internal consistency at all time points (Cronbach's alpha range: 0.672–0.738). Based on the correlation analysis of the two subscales and EPDS, discriminative and convergent validity were indicated at all time points, confirming the construct validity of the Highs scale. Subscale scores showed a significant correlation with EPDS at all time points (*r* = 0.388, 0.384, 0.498, and 0.442, *p* < 0.01).

**Conclusions:** The Japanese version of the Highs scale is reliable and valid, and can be applied for evaluating the hypomanic symptoms during pregnancy and postpartum period.

## Introduction

After the delivery of a child, the incidence of mood disorders is high (1), decreasing the quality of life of postpartum women (2) and leading to a burden on the family (3). It can also result in the abuse of children (4), suicides among postpartum women (5, 6), and negative impact on the growth and development of children through inappropriate upbringing (7, 8). Therefore, it is important to assess mood symptoms and diagnose postpartum mood disorder as early as possible, leading to appropriate treatment.

According to the recent studies, postpartum mood disorder does not first appear during the postpartum period, and in many cases, it is the continuation, or recurrence of mood disorder from pregnancy or the recurrence of mood disorder that existed prior to pregnancy (9, 10). Based on these findings, DSM-IV (11) focused on mood symptoms that occur during 4 weeks after the delivery as postpartum onset specifier, whereas DSM-5 (12) defined the perinatal onset specifier as mood symptoms that occurs during pregnancy and postpartum periods. Therefore, for the early detection of and intervention for mood disorder, the mood states from pregnancy to the postpartum period must be evaluated using the same scale.

The past evaluation scales were developed for assessing the mood symptoms in pregnant and postpartum women. The most commonly used scale is the Edinburgh Postnatal Depression Scale (EPDS) (13). We assessed depressive symptoms by using EPDS and demonstrated that 31.8% of women had experienced depression during pregnancy and 24.8% of women had experienced depression in postpartum period (14). In particular, 42.9% of women with postpartum depression presented with depressive symptoms from pregnancy. In other study, women of identification of depression during 4–6 weeks postpartum. The timing of onset in patients with postpartum depression has been examined, and it has been reported that in 24.9% of women, onset occurs in pre-pregnancy, whereas in 36.7% of women onset is prenatal (15).

Not all women who presented with depressive symptoms suffer from major depressive disorders. Among these women, some may be experiencing depressive states or mixed states as a course of bipolar disorder, but the evaluation of such states in combination with the manic symptoms has been insufficient (16).

At several days postpartum, 9–18.3% of women experience manic symptoms (17, 18). This can be partially explained by the psychological reaction to the event of childbirth. However, among female patients diagnosed with postpartum depression, 13% presented with a hypomanic symptoms in the early postpartum period (19) and some presented with both depressive and hypomanic symptoms in the early postpartum period (17, 20). Given that many women who presented with a manic symptoms in the early postpartum period also presented with a manic symptoms during pregnancy, a manic symptoms during pregnancy or the early postpartum period may be a risk factor for postpartum depression. In addition, among 6.5% of women diagnosed with postpartum depression, the diagnosis was changed to bipolar disorder within 6 months postpartum (21). In patients with bipolar disorder, the cumulative incidence of admission 0–3 months postpartum is 22% (22) and postpartum relapse rates is 37% (23). Bipolar disorders are known to be highly associated with suicide (24). Suicide rates due to perinatal mood disorders are 8.7 per 100,000 births in Japan (25). Among the women who committed suicide during 1 year after delivery, 11% of them suffered from bipolar disorder in perinatal periods (6). Early detection and treatment of bipolar disorders is beneficial for suicide prevention in perinatal periods.

The treatment options differ for depression and bipolar disorder (26). For example, the use of antidepressants for the depressive state or the mixed state in bipolar disorder may lead to the speeding up of the cycle of mood swings and manic states could be evoked (27), treatment resistance could increase (28), and impulsiveness or the risk of suicide could increase (29). Therefore, evaluation of the depressive states and manic states over time, from pregnancy to the postpartum period, is essential for appropriate differential diagnosis and treatment.

The Highs scale is a questionnaire developed by Glover et al. (20) and it evaluates manic symptoms in the first postpartum week. It is a simple screening scale consisting of seven questions that can be quickly answered. Concurrent validity of the Spearman rank correlation of the Highs scale was verified with the Comprehensive Psychopathological Rating Scale (*r* = 0.62) (20) and the Altman Self Rating Mania Scale (*r* = 0.63) (30) in the postpartum period. The internal reliability of the Highs scale has also been also reported at 0.63 (31). Recently, the Highs scale has been used in studies that target not only postpartum but also pregnant women (18); however, whether this evaluation scale can be used throughout pregnancy up to the postpartum period has not yet been examined.

Therefore, we assessed a cohort of pregnant and postpartum women and simultaneously applied the Highs scale and EPDS from pregnancy to the postpartum period to verify the reliability and validity of the Highs scale. In addition, we examined the factor structure of the Highs scale and examined if it is consistent throughout pregnancy and the postpartum period; we examined whether this scale can be used throughout pregnancy and the postpartum period.

## Material and methods

### Participants

Participants in this study were recruited from perinatal classes for pregnant women (starting before the 25th week of pregnancy) at three obstetrical hospitals and one university hospital in central Nagoya, Japan (with a population of ~2 million) between May 2011 and January 2017. Participants were required to be at least 20 years old and capable of understanding the Japanese language.

### Procedures

Pregnant women attending perinatal classes were given detailed information about the study design and methods. This information was given orally and on paper in the four hospitals, and written informed consent was obtained from each participant. Women who agreed to cooperate in the study were asked to complete self-reporting questionnaires, which included social demographic questions, the Highs scale and EPDS in early pregnancy before week 25 (T1), and return them by mail. After receiving the completed consent forms and questionnaires were sent again around week 36 of pregnancy (T2) and returned by mail. At day 5 and 1 month after delivery (T3, T4), the Highs scale and the EPDS were sent and returned by mail.

## Measures

### Highs scale

The Highs scale is a self-rating questionnaire to assess hypomania shortly after delivery and it was developed on the basis of the Schedule for Affective Disorders and Schizophrenia-Lifetime Version (SADS) (20, 32). The Highs scale consists of seven simple questions. The Highs scale is rated on a three-point Likert Scale: “Yes, a lot” (2 points), “Yes, a little” (1 point), or “No” (0 point). The total score ranges between 0 and 14, with cutoff scores between 5 and 8 (20, 30, 33). The high score indicates hypomania. Using a scale threshold of ≥ 8, 100% of the women with hypomania as define by the Highs scale also had hypomania as defined by the Altman Self Rating Mania Scale (AMRS) (30). The Highs scale was poorly correlated with the EPDS and the Kennerley Blues Scale at the postpartum period (17, 20), and fairly correlated with the Comprehensive Psychopathological Rating Scale mania subscale and the AMRS at the postpartum period (20, 30). The internal reliability of the Highs scale has been reported at 0.63 (31).

The Japanese version of the Highs scale was developed by Hasegawa (33). In this process the original version was translated into Japanese. Then, it was retranslated back into English by a native English translator who was unaware of the original wording to confirm that the translation was consistent with the original meaning. Then, it was tested in 119 postpartum women in Japan. The reliability and validity of the Japanese version of Highs scale has not been verified.

### Edinburgh postnatal depression scale (EPDS)

The EPDS is a self-reporting questionnaire designed to assess postpartum depression; it is composed of 10 items scored on a four-point Likert scale. The EPDS have been validated for both postpartum depression (13, 34, 35) and depression during pregnancy (35–38). Numerous studies have used this instrument during pregnancy and the postpartum period. The EPDS Japanese version showed good internal consistency (Cronbach's alpha = 0.78) and test-retest reliability (Spearman's correlation = 0.92) (39). A score ≥9 was designated to screen for minor and major depressive episodes, with a sensitivity of 75 and 82% and a specificity of 93% and 95%, respectively (39, 40).

### Statistical analysis

Participants were randomly divided into two groups. We calculated descriptive statistics for the seven items of the Highs scale. Most of the items were positively skewed (skew values greater than 1) at all time points. We therefore log-transformed all the Highs scale scores for the subsequent factor analysis.

Group 1 was used to conduct for the exploratory factor analysis (EFA) of seven items of the Highs scale. Because all factors were considered dependent upon each other, the factor solution was sought after Promax rotation, which is an oblique rotation. The number of factors was determined by scree plot (41). To create a subscale of the Highs scale, we extracted items for each subscale if they were loaded ≥0.40 on a particular factor but < 0.40 on the other factors.

Group 2 was used to perform the confirmatory factor analyses (CFA) of the factor structure derived from the EFA. The fit of each model with the data was examined in terms of chi-squared (CMIN), degree of freedom (df), comparative fit index (CFI), goodness-of-fit index (GFI), adjusted goodness-of-fit index (AGFI), and root mean square error of approximation (RMSEA).

According to conventional criteria, CMIN/df < 2, CFI > 0.97, GFI > 0.95, AGFI > 0.90 and RMSEA < 0.05 indicate a good fit (42). The Akaike's Information Criterion (AIC) was used to compare different models; a model with an AIC score at least two points lower is regarded as a better model.

As for the combined data of Group 1, Cronbach's alpha for the two hypothesized subscales and total scores were calculated. For examining the evidence of construct validity providing, we also constructed a correlation matrix with the EPDS.

All statistical analyses were conducted using the SPSS version 24.0 and Amos 21.0 (IBM Japan, Tokyo, Japan).

## Results

### Descriptive statistics

A total of 2,435 pregnant women attending the antenatal program were given detailed information about the study design and methods during pregnancy before their consent was obtained. Of the 594 (24.4%) pregnant women who agreed to participate in this study, 461 returned the Highs scale and the EPDS. A total of 418 women (mean age: 32.67 years, standard deviation [*SD*] = 4.60) completed the Highs scale and the EPDS at all four time points. Groups 1 and 2 were consisted of 209 participants each.

Item responses of the Highs scale were shown in Supplementary Table. The mean SDs of all Highs scale items in T1, T2, T3, and T4 were relatively low (Table 1). Most of the Highs scale scores were positively skewed.

**Table 1:** Means and SDs of the Highs scale items at the four time points in Group 1.

**Highs scale items**		**T1**		**T2**		**T3**		**T4**	
		***M (SD)***	***S***	***M (SD)***	***S***	***M (SD)***	***S***	***M (SD)***	***S***
1	More elated than usual	0.33 (0.58)	1.58	0.29 (0.52)	1.57	0.36 (0.58)	1.35	0.21 (0.46)	2.10
2	More active than usual	0.31 (0.56)	1.64	0.31 (0.54)	1.56	0.27 (0.52)	1.85	0.20 (0.44)	1.99
3	More talkative than usual or a pressure to keep on talking	0.17 (0.41)	2.49	0.14 (0.40)	2.93	0.17 (0.43)	2.43	0.12 (0.38)	3.46
4	Thoughts racing	0.31 (0.55)	1.63	0.30 (0.54)	1.59	0.20 (0.47)	2.37	0.22 (0.48)	2.09
5	Feelings of being an especially important person	0.03 (0.19)	6.18	0.03 (0.16)	5.94	0.02 (0.16)	8.26	0.01 (0.12)	8.20
6	Needing less sleep	0.10 (0.35)	3.74	0.23 (0.49)	2.08	0.48 (0.65)	1.03	0.42 (0.59)	1.09
7	Problems with concentrating due to attention jumping to unimportant things	0.30 (0.53)	1.58	0.34 (0.57)	1.47	0.24 (0.49)	1.92	0.33 (0.54)	1.39
	Total	1.55 (1.92)	1.63	1.63 (1.94)	1.47	1.74 (2.14)	1.68	1.51 (1.82)	1.61

*Each item is scored on a three-point Likert scale ranging from 0 to 2. Total scores can range from 0 to 14. M, Mean; S, Skewness; SD, standard deviation; T1, early pregnancy (before week 25); T2, late pregnancy (around week 36); T3, 5 days after delivery; T4, 1 month after delivery*.

### Factor analysis of highs scale items

All the log-transformed items of the Highs scale were entered into the EFA in Group 1. This suggested a two-factor structure (Table 2). The first factor was loaded by three items: “feeling more elated than usual,” “feeling more active than usual,” “feeling more talkative than usual or a pressure to keep on talking.” These items reflected manic symptoms. We named this factor elation. The second factor was loaded by two items: “thoughts racing” and “having problems with concentration due to attention jumping to unimportant things around you.” These items are common to manic and depressive symptoms. We named this factor agitation.

**Table 2:** Factor structure of the Highs scale item over the four time points in Group 1.

**Highs scale items**		**T1**		**T2**		**T3**		**T4**	
		**F1**	**F2**	**F1**	**F2**	**F1**	**F2**	**F1**	**F2**
1	More elated than usual	**0.585**	0.135	**0.871**	−0.030	**0.765**	−0.029	**0.698**	0.071
2	More active than usual	**0.994**	−0.190	**0.658**	0.066	**0.796**	−0.011	**0.771**	−0.182
3	More talkative than usual or a pressure to keep on talking	**0.445**	**0.410**	**0.430**	0.208	**0.554**	0.141	**0.436**	0.346
4	Thoughts racing	0.103	**0.617**	−0.050	**0.988**	−0.031	**1.012**	−0.043	**0.763**
5	Feelings of being an especially important person	0.152	0.157	0.015	0.088	0.283	−0.001	−0.009	0.077
6	Needing less sleep	0.203	0.171	0.097	0.263	0.198	0.104	0.021	0.393
7	Problems with concentrating due to attention jumping to unimportant things	−0.098	**0.636**	0.138	**0.404**	0.105	**0.496**	−0.058	**0.652**

*F1, Factor1; F2, Factor2. Factor loadings≥0.40 are bold*.

The factor structure extracted in the EFA of the Highs scale was then subjected to the CFA using Group 2. The two latent factors were moderately correlated with each other. To examine stability of the factor structure of the Highs scale we conducted a series of CFAs using all the periods.

In T1, item 3 factor loading of Factor 1 and Factor 2 are nearly equal by EFA (Table 2). Then, we examined CFA in two possible model. Model 1 composed Factor 1 (items 1, 2, and 3) and Factor 2 (items 4 and 7). Model 2 composed Factor1 (items 1 and 2) and Factor 2 (items 3, 4, and 7). As a result, the AIC is 24.618 in Model 1, and 30.845 in Model 2. Therefore, we adopted Model 1, which is identical with the model in other time points.

The path values and accompanying fit indices are shown in Table 3. The correlation between Factor 1 and Factor 2 was moderate in all four periods (T1, 0.45; T2, 0.49; T3, 0.52; and T4, 0.36). Examination of CMIN/df, CFI, GFI, AGFI, and RMSEA revealed the current model to meet good fit criteria for all four periods. The fitness of the model to data was good for data from all four periods.

**Table 3:** CFA of the Highs scale items over the four time points in Group 2.

**Highs scale items**		**T1**	**T2**	**T3**	**T4**
F1:ELATION^*^)					
1	More elated than usual	0.66	0.71	0.80	0.72
2	More active than usual	0.74	0.60	0.73	0.76
3	More talkative than usual or a pressure to keep on talking	0.60	0.60	0.71	0.58
**F2:AGITATION** ^*^					
4	Thoughts racing	0.88	0.92	0.86	0.98
7	Problems with concentrating due to attention jumping to unimportant things	0.28	0.35	0.66	0.45
Covariance between F1 and F2		0.45	0.49	0.52	0.36
CMIN/df		0.654	0.867	1.898	0.332
CFI		1.000	1.000	0.988	1.000
GFI		0.995	0.994	0.986	0.997
AGFI		0.981	0.976	0.948	0.990
RMSEA		0.000	0.000	0.000	0.000
AIC		24.618	25.468	29.590	23.328

*CFA, confirmatory factor analysis; T1, early pregnancy (before week 25); T2, late pregnancy (around week 36); T3, 5 days after delivery; T4, 1 month after delivery; F1, Factor 1; F2, Factor 2;*.

* *The figures indicate path values; CMIN/df, chi-squared/degree of freedom; CFI, comparative fit index; GFI, Goodness of Fit Index; AGFI, Adjusted Goodness of Fit Index; RMSEA, root mean square error of approximation; AIC, Akaike's Information Criterion*.

### Internal consistency of the highs scale

As for the combined data of Group 1, internal consistency (Cronbach's alpha) of the three items belonging to Factor1 ranged from 0.696 to 0.758 whereas that of two items belonging to Factor 2 ranged from 0.553 to 0.694, showing reasonable internal consistency. Furthermore, the 5-item Cronbach's alpha ranged from 0.672 to 0.738, whereas the 7-item Cronbach's alpha ranged from 0.664 to 0.695, showing reasonable internal consistency (Table 4).

**Table 4:** Internal consistency (Cronbach's alpha) of the Highs scale subscale and overall scale.

	**F1**	**F2**	**5 items**	**7 items**
T1	0.729	0.553	0.672	0.675
T2	0.724	0.610	0.718	0.694
T3	0.758	0.694	0.738	0.695
T4	0.696	0.630	0.679	0.664

*T1, early pregnancy (before week 25); T2, late pregnancy (around week 36); T3, 5 days after delivery; T4, 1 month after delivery; F1, Factor 1; F2, Factor 2*.

### Construct validity of the highs scale

For examining construct validity, the correlation of the Highs Scale with EPDS was analyzed. Factor 1 was slightly correlated and Factor 2 was moderately correlated with the EPDS scores in all four periods (Table 5).

**Table 5:** Pearson's correlations of the Highs scale subscale scores and EPDS, *n* = 418.

		**T1**		**T2**		**T3**		**T4**		**EPDS**			
		**F1**	**F2**	**F1**	**F2**	**F1**	**F2**	**F1**	**F2**	**T1**	**T2**	**T3**	**T4**
T1	F1	-	0.286^*^	0.361^*^	0.228^*^	0.297^*^	0.066^*^	0.373^*^	0.136^*^	0.179^*^	-	-	-
	F2	-	-	0.220^*^	0.495^*^	0.198^*^	0.277^*^	0.179^*^	0.372^*^	0.388^*^	-	-	-
T2	F1	-	-	-	0.348^*^	0.341^*^	0.154^*^	0.340^*^	0.163^*^	-	0.126^*^	-	-
	F2	-	-	-	-	0.240^*^	0.461^*^	0.196^*^	0.364^*^	-	0.384^*^	-	-
T3	F1	-	-	-	-	-	0.372^*^	0.452^*^	0.264^*^	-	-	0.234^*^	-
	F2	-	-	-	-	-	-	0.164^*^	0.423^*^	-	-	0.498^*^	-
T4	F1	-	-	-	-	-	-	-	0.290^*^	-	-	-	0.182^*^
	F2	-	-	-	-	-	-	-	-	-	-	-	0.442^*^

EPDS, Edinburgh Postnatal Depression Scale; T1, early pregnancy (before week 25); T2, late pregnancy (around week 36); T3, 5 days after delivery; T4, 1 month after delivery; F1,Factor 1; F2,Factor 2;

* *p < 0.01*.

### Correlation among four times point of the highs scale

The correlation between the subscales was analyzed at four time points, and the correlation of subscales between the four time points was confirmed. Pearson's correlations of Highs scale subscales are shown in Table 5. The score of Factor 1 was significantly correlated over the four time points (all *p* < 0.01), and these of Factor 2 was also significantly correlated over the four time points (all *p* < 0.01).

## Discussion

The Highs scale is a self-reporting questionnaire developed to evaluate hypomanic symptoms in the first postpartum week. Given that the correlation with evaluation scales for the postpartum depressive symptom is low, whereas the correlation with evaluation scales for the manic symptom is high, its concurrent validity has been reported (20, 30, 31, 43). However, it has not been clarified whether the reliability and validity of this scale is consistent from pregnancy to the postpartum period. To confirm the measurement invariance, we must confirm that the factor structure is similar from pregnancy to postpartum period. We assessed a cohort of pregnant and postpartum women in this study and applied the Highs scale and EPDS from pregnancy to the postpartum period. This study is the first which evaluated the reliability and validity of the Highs scale, along with whether the factor structure remains the same from pregnancy to the postpartum period.

Our examination of the factor structure for the Highs scale clearly identified that the factor structure with the highest fit for all four time points from pregnancy to the postpartum period was a two-factor structure. Factor 1 consists of items associated with heightened mood and activity, indicating the characteristic symptoms of a manic state (elation). Factor 2 consists of items associated with distractibility and diminished ability to think (agitation). The present results indicated that the Highs scale includes symptoms of elation as well as agitation. In DSM-5, distractibility, together with psychomotor agitation and irritability, is excluded from the symptoms for “with mixed feature” specifier. Some researchers criticized that this may reduce diagnostic power of mixed features of mood disorders (44). Our result indicates that distractibility is associated with racing thoughts, suggesting the importance of psychomotor symptoms in the assessment of pregnant and postpartum women.

Our study indicated that the Highs scale has a two factor structure, consisting of 5 items, and that the two items (item 5 and 6) does not belongs to any factors. The Highs scale was made according to SADS, which is developed to evaluate mood symptoms including severe manic symptoms. An item 5 asks about the symptom of inflated self-esteem or grandiosity, which is often associated with severe hypomania. Then, in our subjects, it is possible that they did not show sufficient factor loading amounts to constitute factors. In addition, although item 6 is a symptom related to sleepless, sleep problems are frequently affected in perinatal women. Therefore, it might not be extracted as symptoms specific to hypomania.

No other studies have examined the factor structure of the Highs scale. In addition, to our knowledge, no other studies have examined the factor structures of manic or hypomanic symptoms in perinatal women. The factor structures of the Young Mania Rating Scale (YMRS) were examined in patients consecutively admitted to an acute psychiatry unit, suggesting that YMRS had the three factors corresponding to “elated mania,” “irritable mania,” and “psychotic mania”(45). The Highs scale was developed to assess hypomanic symptoms in postpartum women. Therefore, unlike in the acute psychiatric patients, we did not identify “psychotic mania.” However, the other two factors were identical to the results of our study. This suggests that the hypomanic symptomatology of perinatal women is similar to hypomania in psychiatric patients.

Previous studies reported that, among the women with positive EPDS findings, some were diagnosed with bipolar disorder during pregnancy (46) and the postpartum period (47), and many who were diagnosed with bipolar disorder experienced depressive episodes (44). In addition, approximately one-third of patients with bipolar disorder have a history of attempted suicide within their lifetime (48), and suicide has become the greatest factor influencing the life prognosis of patients with bipolar disorder. In particular, the agitated symptoms in mixed states are assumed to be a risk factor for suicide attempts and self-harm (49). Furthermore, the use of antidepressants to treat bipolar disorder involve risks such as worsening of agitation, manic switch, and treatment resistance (27–29, 50). By evaluating possible manic states along with depressive states, worsening of agitation or shift to bipolar disorder is detected at an early stage, thereby contributing to appropriate treatment.

When we examined the reliability of the factors of the Highs scale and of the scale itself, sufficient internal consistency was noted. In our study, the 5-item Cronbach's alpha values ranged from 0.672 to 0.738, whereas the 7-item Cronbach's alpha values ranged from 0.664 to 0.695. A scale with a Cronbach's alpha between 0.65 and 0.70 is regarded minimally acceptable (51). Therefore, the Japanese version of the Highs scale was considered to have sufficient reliability. Cronbach's alpha in 5 days after the delivery is higher than that during the pregnancy. This fact is not surprising, because the High scale is developed to evaluate hypomania in the first week after the delivery. However, it can be noted that Cronbach's alpha is almost consistent from pregnancy to postpartum periods.

In addition, the validity of the Highs scale was verified using EPDS, which had been simultaneously used. It was shown that Factor 1 exhibits discriminant validity and Factor 2 exhibits convergent validity, thereby confirming the construct validity of the Highs scale.

The correlation between the two factors of the Highs scale was confirmed during pregnancy and the postpartum period, confirming the association between the subscales. This indicates that the inter-subscale association is stable over time during pregnancy and the postpartum period.

This study confirmed the reliability and validity of the factor structure in the Highs scale during pregnancy and the postpartum period, and this scale was shown to be applicable to evaluate manic states throughout the perinatal period. We confirmed that the Highs scale includes two subscales: elation and agitation. The correlation between “agitation” and EPDS was confirmed, and “agitation” was associated not only with manic states but also with depressive states, suggesting the possible relationship with mixed states. Through evaluation using the same scale from pregnancy to the postpartum period, the continuous observation of mood state fluctuations was possible. In addition to evaluating depressive states using EPDS, the evaluation of manic states using the Highs scale in parallel enabled appropriate diagnosis and treatment selection.

This study had some limitations. This study used self-administered questionnaires without diagnostic procedures based on operational diagnostic criteria. The validity of the Highs scale as a screening test for hypomania can be determined only if the accuracy of the screening test can be compared to the precise diagnosis of hypomania. Therefore, the predictive validity of this scale should be examined in future studies.

In this study, participants of this study were recruited from perinatal classes for pregnant women, and they participated in this study voluntarily. Therefore, this sample may not be representative of the total population. And, it also means that patients in severe depressive and manic states might have been unable to respond to the questionnaire and were lost to follow-up.

For cultural adaptation of the scale, translating the original instrument from one language to another is insufficient; it must be culturally adapted to the target population (52). The Japanese version of Highs scale was developed through blind backward translation and pilot testing in Japanese perinatal women. However, it was not tested in a bilingual sample and full psychometric properties were not compared between English- and Japanese-speaking samples. Therefore, we could not rule out the possibility of differences in linguistic meanings between the original and the translated versions. The response rate of each scale can be influenced by culture and language, and therefore a cultural comparison is also needed in the future.

## Conclusions

The two-factor structure of elation and agitation of the Highs scale is common to both pregnant and postpartum women, indicating that the scale is reliable and valid. The Highs scale can be applied for evaluating the manic state during pregnancy and the postpartum period.

## Data availability

The raw data supporting the conclusions of this manuscript will be made available by the authors upon request.

## Ethics statement

The study was explained to all participants both verbally and in writing, and written informed consent was obtained from each participant. This study protocol was approved by the Ethics Committee of the Nagoya University Graduate School of Medicine, the Ethics Committee of Kaseki Hospital, the Ethics Committee of Nagoya Teishin Hospital and the Ethics Committee of Royal Bell Clinic. The study was conducted in accordance with the established ethical standards of all institutions.

## Author contributions

SM, SG, AK and NO conceived and designed this study. AY, TO, MM, YN, CK, and MO performed this study. AY, MM, YN, CK, and MO contributed reagents, materials and analysis tools. AY, TO, MA, and NO conducted the statistical analysis. AY, TO, and NO wrote the paper. All authors have read and approved the final the manuscript.

### Conflict of interest statement

The authors declare that the research was conducted in the absence of any commercial or financial relationships that could be construed as a potential conflict of interest.
