# Supplementary material for: Validation and Factor Analysis of the Japanese Version of the Highs Scale in Perinatal Women
Source: Front Psychiatry. 2018 Jun 28;9:269. doi: 10.3389/fpsyt.2018.00269 (PMC6032995; doi:10.3389/fpsyt.2018.00269)
Supplement: Supplementary file 1 [file Data_Sheet_1.docx]

**Supplemental Table.** Item responses to the Highs scale, n=418

|  | T1 | | | T2 | | | T3 | | | T4 | | |
| --- | --- | --- | --- | --- | --- | --- | --- | --- | --- | --- | --- | --- |
|  | No | Yes,  a little | Yes,  a lot | No | Yes,  a little | Yes,  a lot | No | Yes,  a little | Yes,  a lot | No | Yes,  a little | Yes,  a lot |
|  | N (%) | N (%) | N (%) | N (%) | N (%) | N (%) | N (%) | N (%) | N (%) | N (%) | N (%) | N (%) |
| Item 1 | 304  (72.7) | 90  (21.5) | 24  (5.7) | 309  (73.9) | 96  (23.0) | 13  (3.1) | 288  (68.9) | 109  (26.1) | 21  (5.0) | 340  (81.3) | 69  (16.5) | 9  (2.2) |
| Item 2 | 309  (73.9) | 88  (21.1) | 21  (5.0) | 306  (73.2) | 96  (23.0) | 16  (3.8) | 323  (77.3) | 79  (18.9) | 16  (3.8) | 339  (81.1) | 73  (17.5) | 6  (1.4) |
| Item 3 | 356  (85.2) | 55  (13.2) | 7  (1.7) | 367  (87.8) | 43  (10.3) | 8  (1.9) | 353  (84.4) | 57  (13.6) | 8  (1.9) | 378  (90.4) | 31  (7.4) | 9  (2.2) |
| Item 4 | 309  (73.9) | 90  (21.5) | 19  (4.5) | 308  (73.7) | 94  (22.5) | 16  (3.8) | 348  (83.3) | 57  (13.6) | 13  (3.1) | 337  (80.6) | 69  (16.5) | 12  (2.9) |
| Item 5 | 405  (96.9) | 12  (2.9) | 1  (0.2) | 407  (97.4) | 11  (2.6) | 0  (0.0) | 410  (98.1) | 7  (1.7) | 1  (0.2) | 412  (98.6) | 6  (1.4) | 0  (0.0) |
| Item 6 | 383  (91.6) | 28  (6.7) | 7  (1.7) | 336  (80.4) | 69  (16.5) | 13  (3.1) | 254  (60.8) | 127  (30.4) | 37  (8.9) | 265  (63.4) | 131  (31.3) | 22  (5.3) |
| Item 7 | 308  (73.7) | 95  (22.7) | 15  (3.6) | 297  (71.1) | 100  (23.9) | 21  (5.0) | 329  (78.7) | 77  (18.4) | 12  (2.9) | 296  (70.8) | 108  (25.8) | 14  (3.3) |

N, sample sizes; T1, early pregnancy (before week 25); T2, late pregnancy (around week 36);

T3, 5 days after delivery; T4, 1 month after delivery
